# On and off signaling pathways in the retina and the visual system

**DOI:** 10.3389/fopht.2022.989002

**Published:** 2022-08-26

**Authors:** Tomomi Ichinose, Samar Habib

**Affiliations:** ^1^ Department of Ophthalmology, Visual and Anatomical Sciences, Wayne State University School of Medicine, Detroit, MI, United States; ^2^ Department of Medical Parasitology, Mansoura Faculty of Medicine, Mansoura University, Mansoura, Egypt

**Keywords:** retina, bipolar cell, parallel processing, visual system, primary visual cortex, superior colliculus

## Abstract

Visual processing starts at the retina of the eye, and signals are then transferred primarily to the visual cortex and the tectum. In the retina, multiple neural networks encode different aspects of visual input, such as color and motion. Subsequently, multiple neural streams in parallel convey unique aspects of visual information to cortical and subcortical regions. Bipolar cells, which are the second-order neurons of the retina, separate visual signals evoked by light and dark contrasts and encode them to ON and OFF pathways, respectively. The interplay between ON and OFF neural signals is the foundation for visual processing for object contrast which underlies higher order stimulus processing. ON and OFF pathways have been classically thought to signal in a mirror-symmetric manner. However, while these two pathways contribute synergistically to visual perception in some instances, they have pronounced asymmetries suggesting independent operation in other cases. In this review, we summarize the role of the ON–OFF dichotomy in visual signaling, aiming to contribute to the understanding of visual recognition.

## 1 Introduction

**Figure 1:**
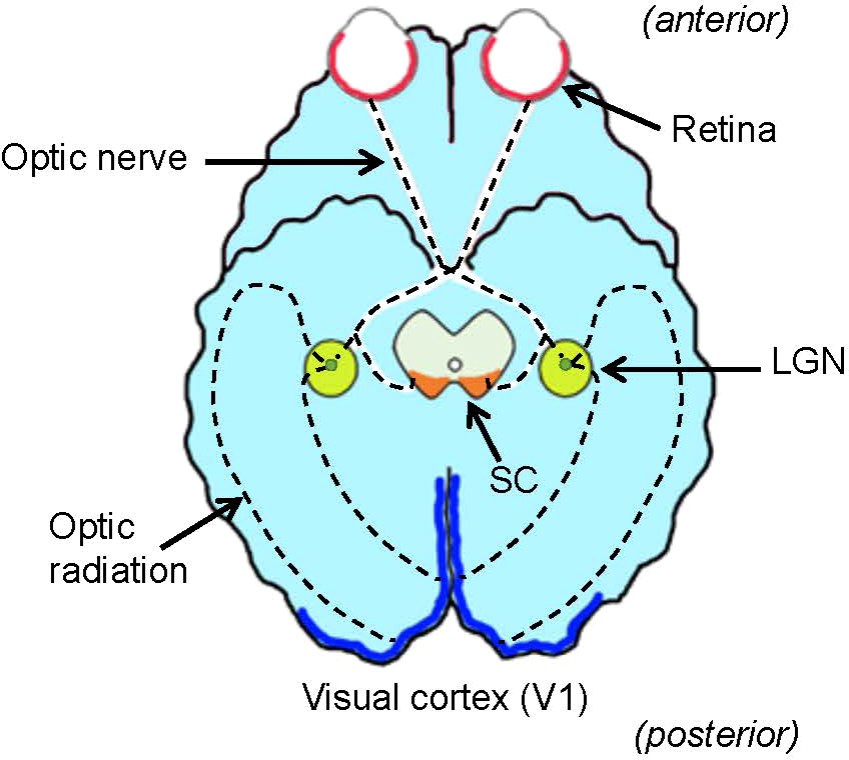
Ventral view of the brain and visual system. The retina (red) in the eye sends visual signals through the optic nerve. The lateral geniculate nucleus (LGN, green) relays visual signals to the visual cortex (V1, blue) in the cerebral cortex. Some signals are also relayed to the superior colliculus (SC, orange) in the midbrain tectum. Dotted lines denote the visual pathway.

Foundational processing of the visual environment occurs in the retina of the eyes, the lateral geniculate nucleus (LGN) in the thalamus, the primary visual cortex (V1), where visual recognition occurs, and the superior colliculus (SC) in the tectum, a center for eye movement (**Figure 1**). Until several decades ago, the function of the retina was thought to involve capturing visual signals, much like a camera film, and then sending them to the brain. Recent evidence has suggested that the retina contains numerous intricate neural networks that analyze the captured visual signals and actively starting visual signal processing. Understanding how the entire visual system conducts visual signal processing is the ultimate goal of vision research. However, the critical centers of the visual system are located far from each other, rendering it difficult to undertake a systematic investigation. This review focuses on a set of classical visual pathways, the ON and OFF pathways, and discusses how they are generated, conveyed, and utilized throughout the visual system.

Visual signal recognition is achieved by parallel processing, that is, multiple features of visual signals are encoded into separate neural streams and analyzed in parallel (1). Parallel processing is initiated by separation of visual signals in retinal neurons, including photoreceptors, bipolar cells, and ganglion cells, and are coded to multiple types of retinal ganglion cells. Each visual pathway is then sent to unique sets of destinations in the brain. In the primate retina, two main pathways have been identified as being involved in parallel processing, namely, the magnocellular pathway, which encodes motion and changes in the visual scene, and the parvocellular pathway, which encodes the shape and color of visual information (2, 3).

**Figure 2:**
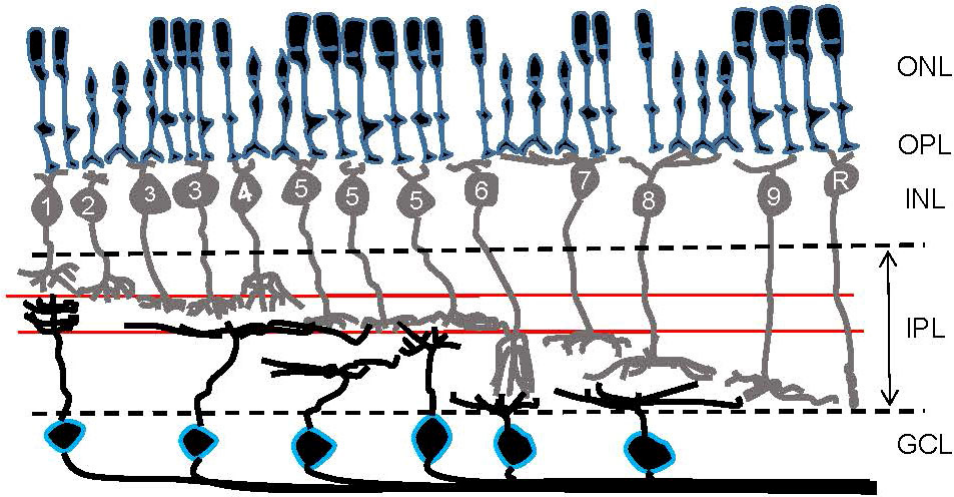
A schematic showing retinal organization. First-order neurons—rod and cone photoreceptors—occupy the outer nuclear layer (ONL). At the outer plexiform layer (OPL), visual signals are transferred to second-order neurons—bipolar cells—in the inner nuclear layer (INL). Numbers are included in bipolar cells’ somas to show their types in the mouse retina. Then, at the inner plexiform layer (IPL), visual signals are transferred from bipolar cells to third-order neurons—ganglion cells—in the ganglion cell layer (GCL). Ganglion cell somas are outlined in blue. Two red lines in the IPL indicate the ChAT bands: OFF ChAT band (upper) and ON ChAT band (lower), which are utilized as a landmark of the IPL.

Parallel processing is initiated at the photoreceptors, the first-order neurons in the retina, where color information is separated by cone photoreceptors with different wavelength sensitivities (4–12). Visual signals are then transmitted from photoreceptors to the second-order neurons, bipolar cells, where signals are further subdivided. Approximately 15 subtypes of bipolar cells have been identified in the retinas of vertebrates, including primates, rodents, fish, birds, and salamanders (**Figure 2**) (13–21). Each type of bipolar cell is thought to extract unique features of visual scenes and initiate distinct parallel signaling pathways. Two well-studied bipolar cell pathways are chromatic signaling pathways (22–25) and ON/OFF-signaling pathways (13, 26, 27).

**Figure 3:**
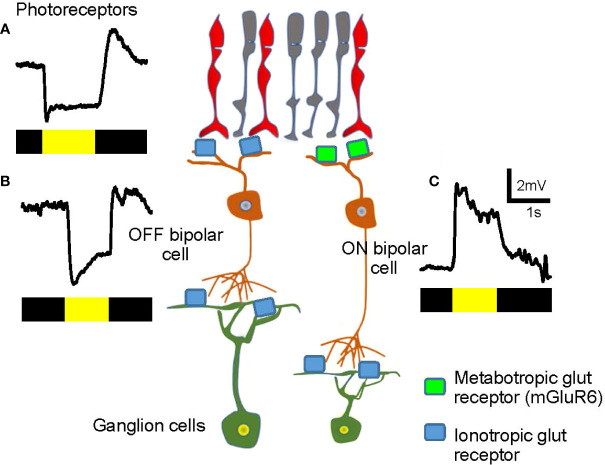
Visual signals in the first- and the second-order neurons in the retina. **(A)** A representative visual signal in photoreceptors. Photoreceptors hyperpolarize when illuminated and depolarize at light offset. The bar color indicates the timing of dark (black) and light (yellow) stimuli. **(B)** An OFF bipolar cell hyperpolarizes when illuminated. The signal is transferred from photoreceptors *via* ionotropic glutamate receptors (light blue) and the sign of the signal is preserved. **(C)** An ON bipolar cell depolarizes when illuminated. The signal is transferred from photoreceptors through metabotropic glutamate receptors (mGluR6, green) and the sign of the signal is inverted.

The ON–OFF separation occurs at the dendrites of bipolar cells (discussed in more detail below). The retina senses the contrast of an object, either bright or dark, against the background luminance. Half of the bipolar cells depolarize in response to a visual signal brighter than the background, whereas the other half hyperpolarizes in response to the bright object, but depolarizes when the illumination ends (**Figure 3**). Cells that respond to the onset of light (or offset of dark) are ON bipolar cells (**Figure 3C**) while cells responding to light offset (or onset of dark) are OFF bipolar cells (**Figure 3B**). Signals in ON and OFF bipolar cells are transferred to ON- and OFF-responding ganglion cells, respectively. A subset of ganglion cells collects both ON and OFF signals, which are described as ON-OFF ganglion cells. Then, ON, OFF, and ON-OFF ganglion cells send their signals to a unique set of targeted neurons in the LGN and other parts of the brain to achieve visual perception.

Separated ON and OFF visual signals in the retina are conveyed through independent parallel processing neural streams to the brain where ON and OFF signals start to combine. Because ON and OFF signaling pathways convey the light and dark signaling of the same object, it has been classically thought to signal in a mirror-symmetric manner. However, asymmetric ON and OFF signaling has been reported in the retina and V1. Furthermore, ON and OFF signal coordination outlines the features of the object for some object recognition (*e.g*. orientation tuning and direction selectivity), whereas ON and OFF signals separately operate to achieve the object recognition for other object recognitions (*e.g.* optokinetic reflex (OKR) and looming stimulus-evoked fear responses). In this review, we focus on ON–OFF signaling to explore how the visual system executes the visual recognition process.

## 2 On and off signaling in the visual system

ON and OFF signaling pathways are separated at the retinal photoreceptor-bipolar cell synapse. The separated signals are transmitted to amacrine and ganglion cells in the retina, which are transferred to LGN neurons. The separated ON and OFF signaling streams are subsequently integrated into the visual cortex and superior colliculus (SC).

### 2.1 Retina

Both rod and cone photoreceptors hyperpolarize to light and depolarize under dark conditions (**Figure 3A**). In the dark, photoreceptors are depolarized and continuously release the neurotransmitter glutamate, thereby providing signals to subsequent neurons, the bipolar cells. OFF bipolar cells possess ionotropic glutamate receptors and depolarize in the dark in response to glutamate released by the photoreceptors (27–31). In contrast, ON bipolar cells express the metabotropic glutamate receptors, mGluR6, on their dendrites. mGluR6 links to the G protein, Go, and consequently, the downstream second-messenger system, which leads to the cell hyperpolarization following glutamate binding (32, 33). Therefore, ON bipolar cells hyperpolarize in the dark, which is the opposite sign of photoreceptors and OFF bipolar cells.

When the retina is illuminated, photoreceptors hyperpolarize and reduce their glutamate release.ON bipolar cells are free from the suppressive effect of glutamate binding to mGluR6, resulting in activation of the mGluR6-linked cation channel, TRP M1, to depolarize the bipolar cell (**Figure 3C**) (34–36). In contrast, reduced glutamate release from photoreceptors leads to hyperpolarization of OFF bipolar cells (**Figure 3B**). In brief, ON bipolar cells hyperpolarize in the dark, which is the opposite of that seen for photoreceptors and OFF bipolar cells. In response to light, ON bipolar cells depolarize, and OFF bipolar cells hyperpolarize. Accordingly, bipolar cells are the site of ON and OFF signaling separation.

The ON and OFF dichotomy is received by downstream amacrine and ganglion cells. Signal transfer occurs in the inner plexiform layer (IPL), where ON and OFF cells ramify in separate layers (**Figure 2**). The outer half of the IPL is the OFF-signaling layer and is where OFF bipolar, amacrine, and ganglion cells make synaptic connections, while the inner half of the IPL is where ON bipolar, amacrine, and ganglion cells extend their processes for the synaptic transmission of ON signaling. Dozens of ganglion cell types have been recently identified in the retinas of mice (37, 38) and primates (39, 40), with most being categorized as either ON, OFF, or ON-OFF ganglion cells based on their ramification patterns in the IPL. Retinal ganglion cells extend their axons to the brain forming the optic nerve, through which the signals are transmitted.

ON and OFF bipolar cells innervate ganglion cells that include dozens of types. Many types of ganglion cells possess ON and OFF counterparts, such as ON brisk-transient and OFF brisk-transient ganglion cells. Ravi et al. (41) recorded spikes from ON and OFF pairs of brisk-sustained, brisk-transient, and small transient ganglion cells and found asymmetries between ON and OFF signals. ON brisk-sustained cells possess a larger receptive field and respond briefer than their OFF counterparts. In contrast, ON brisk-transient ganglion cells possess a smaller receptive field and respond more sustained than their OFF counterparts, and ON and OFF small-transient ganglion cells have similar spatial and temporal profiles. Asymmetries in temporal, spatial, and linearity characteristics between ON and OFF ganglion cells of the same types have been reported in the mouse retina (42, 43) and the macaque retina (44, 45).

The ON and OFF asymmetry in temporal aspects probably does not arise due to the different glutamate receptors present in OFF and ON bipolar cells because in the mammalian retina, there are no significant temporal differences between ON and OFF bipolar/ganglion cells (29, 37, 44, 46). Instead, the ON and OFF asymmetry is in a ganglion cell type-dependent manner, suggesting that the combination of bipolar cell inputs and amacrine cell inputs to a type of ganglion cells shapes the asymmetry.

### 2.2 Lateral geniculate nucleus to the primary visual cortex

#### 2.2.1 Lateral geniculate nucleus

Many types of ganglion cells project to multiple locations in the central nervous system. The lateral geniculate nucleus (LGN) is a major target for ganglion cells (**Figure 1**), which is divided into dorsal and ventral parts. Image-forming ganglion cells target the dorsal LGN (dLGN), relaying visual signals from the retina to the visual cortex (47–49). In contrast, non-image-forming ganglion cells, including the intrinsic photosensitive retinal ganglion cells (ipRGCs), project to the ventral LGN (50, 51). These observations indicate that visual stimulation-evoked responses take place in the dLGN.

In the dLGN, parvocellular and magnocellular ganglion cells from ipsilateral and contralateral eyes project into multiple distinct layers. The ON–OFF dichotomy is preserved in each region of the dLGN (52), suggesting that the mixing of ON/OFF signals does not occur in the thalamic structure. Koniocellular LGN neurons exhibit transient combined ON–OFF responses, which may originate from broad-thorny ON–OFF retinal ganglion cells (52) and not from separate ON and OFF ganglion cells. This case also suggests that ON and OFF signaling is relayed as transferred from the retina.

In the mouse and rat dLGN, color-responsive cells exhibit ON and OFF dichotomy (53, 54). Chromatic cells show color opponency with opposite ON and OFF responses when activated by green or UV light. In the mouse retina, cone photoreceptors contain two types of opsins, green and UV (55), which are transferred to green-OFF (Type 1) and UV-ON (Type 9) chromatic bipolar cells (9, 25). Concomitantly, some types of ON ganglion cells exhibit color opponency in a region of the retina, with green light evoking OFF responses and UV light triggering ON responses, consistent with the signs of chromatic bipolar cells (56). Most color-opponent cells in the dLGN show the same color response signs, also suggesting that dLGN neurons receive ON and OFF signals from retinal ganglion cells and relay the signals to the subsequent neurons without mingling the signals.

#### 2.2.2 Primary visual cortex (V1)

**Figure 4:**
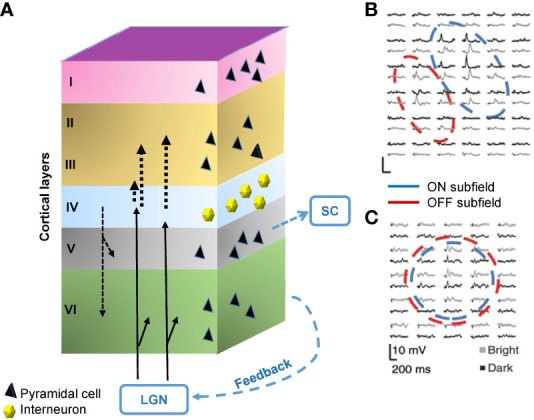
Structure and the signals of the primary visual cortex (V1). **(A)** The V1 comprises six layers of neurons, including pyramidal cells and interneurons. The visual signal from the lateral geniculate nucleus (LGN) innervates Layer 4 and partially also Layer 6. Layer 4 interneurons relay signals to Layer 2/3. There are also interlayer connections, feedback connections to the LGN, and feedforward connections from Layer 5 to the superior colliculus (SC). **(B)** A representative receptive field of a simple cell in Layer 4, which contains aligned and elongated ON and OFF subfields. Black and gray traces show per-stimulus time histograms measured with dark and light stimuli, respectively. **(C)** A representative receptive field of a complex cell in Layer 2/3. The ON and OFF subfields show greater overlap. **(B)** and **(C)** are adapted with permission from Martinez et al. (61).

The ON and OFF pathways are still separated in the dLGN, which are conveyed and integrated into the primary visual cortex (V1). The V1 is composed of six layers of cytoarchitecture. Thalamocortical fibers entering area V1 from the dLGN project mainly to Layer 4 but also partly to Layer 6. Layer 4 fibers primarily project to Layer 2/3 in the visual cortices of cats, monkeys, and tree shrews (57–60). In addition to the thalamocortical inputs, neurons make feedforward and feedback connections among layers. Furthermore, there is also considerable feedback input to the LGN and projection to the SC from Layer 5 (**Figure 4A**).

The V1 contains both simple cells and complex cells. Simple cells are mainly found in Layer 4 and receive input through the thalamocortical fibers (62). The receptive fields of many simple cells have adjoining, elongated ON and OFF subfields (**Figure 4B**) (61). This elongated shape differs from the shape of receptive fields of the dLGN and retinal ganglion cells, which show a concentric ON or OFF center with an antagonistic surround. The different shapes are attributable to the convergence of thalamocortical inputs; multiple thalamic neurons in line innervate one simple cell in V1 (62). Complex cells reside outside of Layer 4, which are innervated by simple cells (61, 62), and have greater ON and OFF signals overlap (**Figure 4C**) (61).

ON and OFF signals in V1 are transferred from the dLGN in parallel. Interestingly, these two inputs appeared to be asymmetric in terms of spatial and temporal aspects. In the temporal aspect, the OFF pathway is faster than the ON pathway in cat V1 (63, 64). In line with this, a large, long-lasting stimulus evokes stronger ON than OFF responses, whereas the opposite is seen when a small, fast stimulus is applied (65). The origin of the ON/OFF temporal asymmetry might be originated in the retina, but it has not been fully understood. The ON/OFF temporal difference indicates that dark signals are recognized faster than the light signals.

ON/OFF asymmetry is also observed in the mouse visual cortex. OFF responses dominate in the central visual field where binocular innervation occurs. In contrast, ON and OFF responses are more balanced in the periphery (66).

In the spatial aspect, ON/OFF asymmetry is also detected in the size of receptive field and neuronal linearity/nonlinearity in the LGN and V1 of cats (67) and humans (68). OFF-center cells dominate the areas in V1 of cats (69) and macaque V1 (70, 71), which facilitates the contrast discrimination in the natural scene (72). These features would cause dark stimuli to drive cortical neurons more strongly than light stimuli at low spatial frequencies.

### 2.3 Superior colliculus

**Figure 5:**
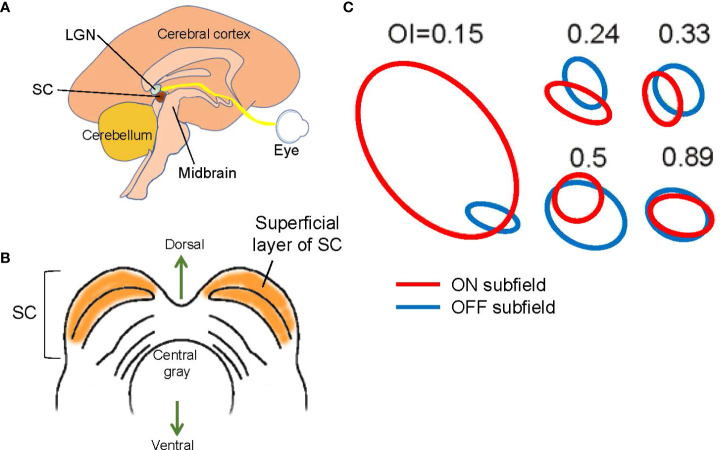
Structure and signals of the superior colliculus (SC). **(A)** A schematic showing a mid-sagittal view of the brain and the locations of the lateral geniculate nucleus (LGN) and the SC. The SC is located in the midbrain tectum. **(B)** A schematic showing a midbrain section at the level of the SC. The superficial layer of the SC is purely visual. **(C)** Examples of SC receptive fields. ON (red) and OFF (blue) subfields show overlap with a range of overlap index (OI). Adapted with permission from Wang et al. (73).

The SC is a midbrain structure and the integrating center for eye movements (**Figures 1**, **5**). Although the SC communicates with the motor, auditory, and visual systems, the superficial layers of the SC are purely visual (**Figure 5B**). Retinal ganglion cells directly project their axons to this region, which preserves retinotopic organization (74, 75). Approximately five to six ON and OFF ganglion cells provide synaptic inputs to one SC cell in mice (76, 77).

Most SC cells are ON and OFF responding (73, 78). Receptive field mapping showed that most SC neurons have spatially overlapping ON and OFF subfields (**Figure 5C**) (73), indicating that the corresponding ganglion cells are neighboring ON and OFF ganglion cells. Interestingly, one study indicated that when the visual cortex is removed, the ON and OFF overlap ratio is decreased (73), suggesting that the corticocollicular inputs, which connect from V1 cortex to the SC, adjust the receptive fields of SC cells. The corticocollicular pathway originates in Layer 5 of V1, where most cells are complex cells with overlapping ON and OFF receptive fields (**Figure 4**). The high ON and OFF overlapping in V1 Layer 5 presumably increases the ON and OFF overlap ratio in SC cells. This observation also suggests that separate ON and OFF ganglion cells, and possibly also some ON-OFF ganglion cells, provide synaptic inputs to single SC cells.

## 3 Unified on and off functions

ON and OFF signals are separated in the retina and integrated into V1 cortex and SC. In this section, we address how the ON and OFF signals contribute to specific visual functions, focusing on two visual functions that result from the synergistical contributions of ON and OFF signals. In the following section, we introduce two visual functions that are achieved by signaling independently through either ON or OFF pathway.

### 3.1 Orientation tuning

**Figure 6:**
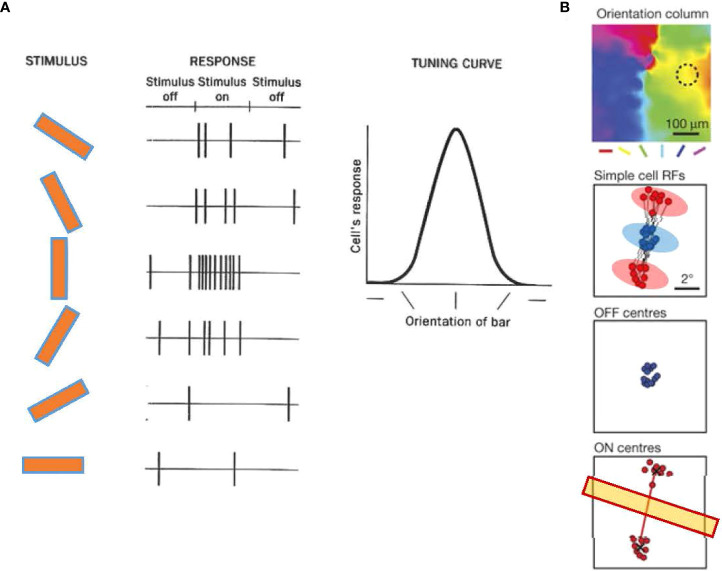
Orientation tuning in cortical neurons of the primary visual cortex (V1). **(A)** Orientation tuning exhibited by a simple cell in cat V1. The cell responded with spiking to a vertical bar. The response decreased when the bar angle became more horizontal, showing that the cell was tuned to a vertical angle orientation. Adapted with permission from Hubel and Wiesel (79). **(B)** A representative simple cell receptive field from and orientation column in tree shrew V1, which shows ON (red) and OFF (blue) subfields. An OFF subfield is flanked by two ON subfields. The angle of the two ON subfields determines the tuning orientation (yellow bar). Adapted with permission from Lee et al. (81).

Several decades ago, Hubel and Wiesel identified the orientation column in the V1 of cats (79, 80). An orientation column is a unit of functional architecture of V1 in which neurons in a columnar region (a small area that encompasses Layer 1 through Layer 6) strongly respond to a bar of a particular orientation (**Figure 6A**). Neurons in neighboring columns show slightly different angle preferences, while neurons in a larger area exhibit preferences to all angles (**Figure 6B**). The orientation preference is determined by the shape of the receptive field of the simple cell in the column, which is elongated and responds to a bar that fits the angle (**Figure 4B**). In addition to cats, functional columnar structure and orientation columns are also present in the V1 of primates (82, 83), tree shrews (84, 85), and ferrets (86).

Recent evidence has shown that the receptive fields of V1 single cells contain ON and OFF subfields, and the arrangement of these subfields is a crucial determinant of the preferences of orientation columns. In the V1 of the tree shrews, the receptive field of a single cell often contains a central OFF subfield flanked by two ON subfields, and the arrangement of the two ON subfields predicts the orientation preference of the column (**Figure 6B**) (81, 87). A similar arrangement is observed in simple cells of the cat V1, which exhibit elongated ON and OFF subfields aligned according to their orientation preferences (88, 89). These observations suggest that ON and OFF cells coordinate to generate the orientation tuning.

In contrast to the cortices of primates and other mammals, the orientation column is not well developed in the V1 of some species, including rabbits and mice (90, 91). Mouse V1 simple cells have ON and OFF subfields within the receptive field; however, there is a lack of identifiable orientation columnar structure (90). Interestingly, in both mice and rabbits, the orientation-selective ganglion cells in the retina appear to be responsible for generating orientation tuning (92–94). Their signs are almost exclusively ON-responding; however, the OFF pathway might generate their antagonistic receptive fields. The orientation tuning mechanism seems to differ between rodents and primates/cats, but the arrangement of both ON and OFF pathways is crucial in all species examined to date.

### 3.2 Direction selectivity

**Figure 7:**
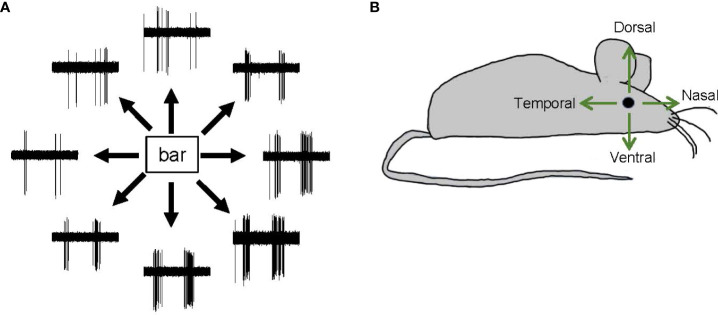
Direction-selective responses and tuning directions. **(A)** Representative spike responses from a retinal ON–OFF direction-selective ganglion cell in response to a bar moving in eight directions. This ganglion cell responded with significant spiking to a bar moving in a bottom-right direction but responded less to a bar moving in a top-left direction. **(B)** A schematic showing the four cardinal directions—dorsal, ventral, nasal, and temporal.

Direction selectivity is a form of motion detection that is first coded in direction-selective ganglion cells (DSGCs) in the retinas of many vertebrates, including mice, fish, and rabbits (48, 74, 95–97). Starburst amacrine cells (SACs), which release GABA onto DSGCs asymmetrically, shape the direction-selectivity of DSGCs (98, 99). DSGCs generate spikes in response to the movement of an object in a specific direction. Each DSGC senses one of the cardinal directions, namely, dorsal, ventral, nasal, or temporal (**Figure 7**). DSGCs display ON, OFF, or ON–OFF signs. However, the existence of DSGCs in the primate retina remains controversial, and there is both evidence for (100, 101) and against (102, 103) this possibility.

The SC is a critical target for retinal direction selectivity, which has been reported in SC cells of many species, including fish, mice, and monkeys (73, 97, 104–106). The receptive field of SC cells contains several ganglion cells with ON, OFF, and ON-OFF signs. Most SC cells respond to both the onset and offset of visual stimuli: ON-OFF responding cells (77, 78).

The ON and OFF signs of retinal DSGCs are species-dependent. ON, OFF, and ON-OFF DSGCs are found in the mouse retina (37); ON and ON-OFF DSGCs but not OFF DSGCs are found in the rabbit retina (107, 108); and ON and OFF DSGCs but not ON-OFF DSGCs are present in the fish retina (109, 110). However, single SC cells receive both ON and OFF signals and show ON-OFF responses (110), suggesting that the motion signal, and not the polarity of contrast (darkness or brightness) is essential information for SC cells.

SC cells and retinal ganglion cells also display differences in directional preference. In the fish retina, DSGCs show three directional preferences, namely, dorso-ventral, ventro-dorsal, and caudo-rostral. However, in the fish tectum of the midbrain, SC neurons also show a rostro-caudal directional preference (106). Similarly, in the mouse retina, DSGCs exhibit only one of the four cardinal directional preferences (dorsal, ventral, nasal, or temporal) (**Figure 7**), whereas SC neurons exhibit a full range of preferred directions (104). Notably, SC neurons receive feedback and feedforward inputs from multiple regions of the central nervous system, and these may be the source of the additional directional preference.

In summary, SC neurons preserve the orientation of the retinotopic map, hold both ON and OFF signals from the retina in their receptive fields, and exhibit unique preferred directions. These properties indicate that SC neurons code the spatial extent and direction of a moving object to initiate smooth pursuit or foveation. For direction selectivity in the SC, retinal ON and OFF cells are the contrast detectors, perceiving the direction of both bright and dark moving objects.

## 4 Independent on and off functions

### 4.1 Optokinetic reflex

**Figure 8:**
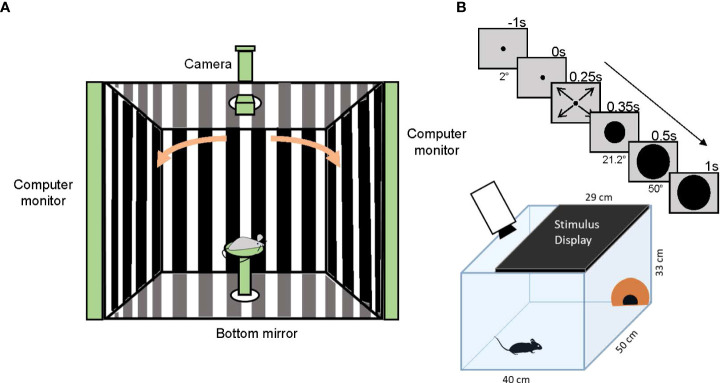
Optomotor reflex and looming-stimulus experiments. **(A)** A schematic showing the optomotor reflex testing device. A mouse is placed on a stage in the middle of the enclosure and observes moving gratings shown on monitors. If the mouse sees the gratings, it chases the motion by moving its head. The camera on top of the enclosure captures an image of the mouse head and the experimenter judges whether the mouse can see the grating movement. **(B)** A schematic showing the looming-stimulus and mouse behavior experiments. A mouse is placed in an arena with a shelter (orange hut). A stimulus display is placed on the arena ceiling. After acclimation, the monitor shows a rapidly expanding black circle (upper panels). The mouse shows a fear response, either dashing to the hut (flight) or freezing.

The optokinetic reflex (OKR) is a visual reflex that helps most vertebrates stabilize retinal images in relation to movements in the visual world. OKR testing comprises slow eye-tracking of the moving vertical stripes in a rotating grating followed by a rapid saccade in the opposite direction, which returns the eye to its primary position (**Figure 8A**) (111). The OKR test is utilized to measure visual acuity and contrast sensitivity in laboratory animals (112), and different models of optomotor devices have been developed to obtain reliable optokinetic responses (113, 114).

Visual kinetic information is sensed by retinal motion detectors; SACs and DSGCs. Detected signals are transmitted through the optic nerve and the optic tract to the visual cortex and SC. The accessory optic system (AOS) contributes to the pathway and receives afferent connections from the nuclei of the optic tract (NOT), the dorsal terminal nucleus (dTN), and the LGN. Efferent connections of the AOS target the brainstem and cerebellar nuclei, which activate the motor neurons of the extraocular muscles (115–117). Using functional ultrasound imaging of the whole brain, Mace et al. (118) identified 87 brain regions that exhibit changes in activity in relation to the OKR.

Some evidence have shown that OKR depends exclusively on the ON pathway and not the OFF pathway. A zebrafish mutant, *no optokinetic response c* (*nrc*), lacks an OKR (119), of which processes invaginated into the photoreceptor terminals and the ribbons in most photoreceptor pedicles appeared to be free-floating in the cytoplasm, indicating that the synaptic connections between photoreceptors and ON bipolar cells were disrupted. Additionally, Emran et al. (120) reported that most of the cells in *nrc* mutants display an OFF function and no pure ON ganglion cells could be detected based on electroretinogram (ERG) and single-unit recordings, and pharmacological blockade of ON pathway in wild-type zebrafish mimicked the *nrc* mutant phenotype.

A study was done by Aung et al. (121) on *nob* and *Vsx1^-/-^* mice which have dysfunctional ON, and partial dysfunction in OFF pathways, respectively. They used spatial frequency and contrast sensitivity thresholds to assess the OKR and reported that both mutants exhibited reduction in OKR. However, the findings noticed in *Vsx1^-/-^* mice were significantly lower than those observed in *nob* mice. This is another evidence that ON pathway is crucial for the OKR, while OFF pathway contributes minimally to the reflex.

Joly et al. (122) used ischemia/reperfusion (I/R) to identify the direction-selective circuits linked to the OKR in mice. Ligation of the central ophthalmic artery and vein for 60 minutes caused irreversible loss of the OKR. Histological analysis revealed that ON SACs and ON DSGCs were markedly affected by I/R, whereas OFF SACs were only minimally impacted. Specifically, notable dendritic loss was observed in the DSGCs at DSGC-ON SAC synaptic connections. The results of this study also showed that the ON pathway is critical for the OKR.

Interestingly, *Drosophila* model exhibited that both ON and OFF pathways are necessary to evoke OKR, while photoreceptor input to either ON or OFF pathway alone is sufficient to sense the moving grating. This paradox is attributable to the separation of photoreceptors output into ON and OFF channels in the lamina and the electrical coupling happening between both pathways which enables cross activity, so a single pathway is activated when only its counterpart is receiving the input (123).

### 4.2 Looming-evoked fear response

The neural pathways responsible for fear responses have recently been explored. When a dark object suddenly approaches, animals react to the object by quickly escaping from it or freezing. The looming stimulus of a rapidly-expanding black disk displayed on a ceiling monitor mimics the approaching dark object, and induces a freezing or rapid flight response in mice (**Figure 8B**) (124, 125). Therefore, the looming stimulus can be used as a vision test if a mouse sees a black object and responds with escaping or freezing.

Because an expanding dark spot with moving edges evokes fear responses, OFF direction-selective cells in the retina are expected to contribute to looming-evoked behavior (124). Two types of OFF cell pathways have been shown to convey looming-evoked fear responses in mice: the PV-5 OFF ganglion cells (126), and a neural pathway of VG3 amacrine cells - W3 & OFF t-α ganglion cells (127, 128). The VG3 (vesicular glutamate transporter 3) cells are one of more than 60 types of amacrine cells that contain glutamate as their neurotransmitter. W3 and OFF t-α cells are types of ganglion cells, which receive synaptic inputs from the VG3 amacrine cells.

VG3 cells depolarize to looming stimuli and hyperpolarize to receding stimuli, leading to the unique innervation of two types of ganglion cells, W3 and OFFα. VG3 cells respond at the onset of motion by transient excitation followed by sustained inhibition, with amplitudes proportional to the speed of the stimulus. W3 cells receive a similar excitation and inhibition pattern and are believed to signal the onset of the looming stimulus. Meanwhile, OFFα ganglion cells respond during looming, and their excitation corresponds to the speed of the stimulus. Therefore, slight delays between excitation and inhibition of both W3 and OFFα ganglion cells inform the brain regarding the onset and speed of stimulus, respectively. The ablation of these neurons eliminates the defensive responses to looming stimuli (127, 128), indicating that the pathway of VG3-W3/OFFα ganglion cells is critical for looming stimulus-evoked fear responses.

Looming-evoked fear responses are induced in both vertebrates and invertebrates. In the fly eye, a looming detector has been identified, named type II (LPLC2) neuron in the lobula plate/lobula columnar (129), which is analogous to the SC of vertebrates (130). These cells respond strongly to a looming stimulus, but weakly to a moving stimulus in a lateral direction. Similarly, in the locust eye, the lobula giant movement detector (LGMD) and its downstream neurons are designated looming detectors (131, 132). These cells respond only to dark, indicating that the looming responses are mediated only by OFF-responding neurons.

## 5 On-off polarity switch

Recent evidence suggests that the retinal neural hardware is more flexible than was previously thought (133). Rivlin-Etzion and her group (134) reported that in ON SACs, a certain light stimulus switched the status of light-evoked responses from depolarized to hyperpolarized. Because SACs are a crucial component of direction selectivity in the retina, the polarity switch in the SACs changed the direction selectivity in DSGCs (135, 136). Additionally, Tikidji-Hamburyan et al. (137) and Pearson and Kerschensteiner (138) found that ON and OFF spikes in ON-OFF ganglion cells were differently generated over a broad range of ambient light levels from scotopic to photopic conditions, resulting in the switching of the ON-dominance and OFF-dominance at different ambient light conditions. Furthermore, Sagdullaev and Mccall (139) and Farrow et al. (140) reported that a bright full-field light stimulus altered ON and OFF polarity in some ganglion cells, a result that was attributable to wide-field amacrine cell inputs that convey signals to distant regions within the retina. Pang et al. (141) and Hoshi et al. (142) suggested that glutamatergic AMPA receptors in ON bipolar cells may also induce cross excitation. Together, these reports provide evidence of a polarity switch between ON and OFF signs in the retina.

The ON and OFF sign switch might be a crucial mechanism for adapting to the saccadic image shift (143, 144). Additionally, the switch might play a role in coding a complicated natural scene (145, 146). How the ON and OFF polarity switch in the retina affects the central projection has not been explored, and future studies should focus on elucidating the ON and OFF pathways and signals in a broad range of visual environments.

## 6 Clinical conditions

Vision disorders in humans regarding the ON and OFF pathways have been reported. Sieving et al. (147) reported that a human patient with unilateral cone dystrophy exhibited an abnormal electroretinogram (ERG) with a sustained positive plateau instead of a standard transient b-wave. The patient’s ERG is similar to a photopic macaque ERG when ionotropic glutamate receptors were blocked pharmacologically, indicating that the OFF pathway is disrupted in this patient. The major complaint of cone dystrophy, including this patient, is decreased color vision (147, 148), which might be originated from the cone dysfunction and the reduced OFF pathway signaling might not be related to the symptom. This is a rare disorder, and physio-pathological mechanisms have not been fully understood.

Vision disorder with a loss of ON pathway has been reported as the congenital stationary night-blindness (CSNB) (149). ERG for these patients shows a reduced or abolished scotopic b-wave, which indicates that the signal transmission from rod photoreceptors to rod bipolar cells, scotopic ON signaling, is disrupted. These patients complain of night blindness, but many of them show normal vision in the daytime. There is a broad spectrum for this disorder; the complete form CSNB patients show the eliminated scotopic b-wave but normal cone photoreceptor functions, whereas the incomplete form CSNB patients exhibit reduced scotopic b-wave and reduced cone functions. Pathological mechanisms include the dysfunction of mGluR6-TrpM1 complex and Ca^++^ channel disorders (150, 151).

## 7 Conclusion

Although the visual system is complicated and only poorly elucidated, it is increasingly accepted that parallel processing represents a crucial mechanism for visual recognition. The ON/OFF dichotomy is generated in the retina through the sensing of the contrast of an object against the background luminance. ON and OFF signaling synergistically or independently conveys the features of the object. Specifically, orientation tuning and direction selectivity are achieved by the interaction between ON and OFF pathways, while the OKR and looming-evoked fear responses are mediated by either ON or OFF pathways independently. This suggests that visual recognition through the cerebral cortex utilizes both pathways, but reflexes use only one of them to promptly respond to a visual scene. Studying how the signals are subsequently transferred to the visual system is crucial for understanding visual signal processing.

## Author contributions

TI designed the article, and TI and SH wrote the manuscript. All authors contributed to the article and approved the submitted version.

## Funding

The present study was supported by the National Institutes of Health (NIH) project grants (R01 EY028915; R01 EY032917), and by Research to Prevent Blindness (RPB).

## Acknowledgments

We thank Mr. Jeremy Bohl for critically reading the manuscript and Dr. Reece Mazade for valuable and insightful suggestions.

## Conflict of interest

The authors declare that the research was conducted in the absence of any commercial or financial relationships that could be construed as a potential conflict of interest.

## Publisher’s note

All claims expressed in this article are solely those of the authors and do not necessarily represent those of their affiliated organizations, or those of the publisher, the editors and the reviewers. Any product that may be evaluated in this article, or claim that may be made by its manufacturer, is not guaranteed or endorsed by the publisher.
